# Improvement of Myelopoiesis in Cyclophosphamide-Immunosuppressed Mice by Oral Administration of Viable or Non-Viable *Lactobacillus* Strains

**DOI:** 10.3389/fimmu.2021.647049

**Published:** 2021-04-12

**Authors:** Andrés Gramajo Lopez, Florencia Gutiérrez, Lucila Saavedra, Elvira Maria Hebert, Susana Alvarez, Susana Salva

**Affiliations:** ^1^ Laboratory of Immunobiotechnology, Reference Centre for Lactobacilli (CERELA-CONICET), San Miguel de Tucumán, Argentina; ^2^ Institute of Applied Biochemistry, Tucumán University, San Miguel de Tucumán, Argentina

**Keywords:** myelopoiesis, lactobacilli, immunobiotic, cyclophosphamide, non-viable *Lactobacillus*

## Abstract

Myelosuppression is the major dose-limiting toxicity of cancer chemotherapy. There have been many attempts to find new strategies that reduce myelosuppression. The dietary supplementation with lactic acid bacteria (LAB) improved respiratory innate immune response and the resistance against respiratory pathogens in immunosupressed hosts. Although LAB viability is an important factor in achieving optimal protective effects, non-viable LAB are capable of stimulating immunity. In this work, we studied the ability of oral preventive administration of viable and non-viable *Lactobacillus rhamnosus* CRL1505 or *L. plantarum* CRL1506 (Lr05, Lr05NV, Lp06V or Lp06NV, respectively) to minimize myelosuppressive and immunosuppressive effects derived from chemotherapy. Cyclophosphamide (Cy) impaired steady-state myelopoiesis in lactobacilli-treated and untreated control mice. Lr05V, Lr05NV and Lp06V treatments were the most effective to induce the early recovery of bone marrow (BM) tissue architecture, leukocytes, myeloid, pool mitotic and post-mitotic, peroxidase positive, and Gr-1^Low/High^ cells in BM. We selected the CRL1505 strain for being the one capable of maintaining its myelopoiesis-enhancing properties in its non-viable form. Although the CRL1505 treatments do not modify the Cy ability to induce apoptosis, both increased the incorporation of BrdU in BM cells. Consequently, Lr05NV and Lr05V treatments were able to promote early recovery of LSK cells (Lin^-^Sca-1^+^c-Kit^+^ cells), multipotent progenitors (Lin^-^Sca-1^+^c-Kit^+^CD34^+^ cells), and myeloid cells (Gr-1^+^Ly6G^+^Ly6C^-^ cells) with respect to the untreated Cy control. In addition, these treatments were able to increase the frequency of IL17A-producing innate lymphoid cells in the intestinal lamina propria (IL-17A^+^RORγt^+^CD4^-^NKp46^+^ cells) after Cy injection. These results were correlated with an increase in the IL-17A serum levels, a GM-CSF high expression and a CXCL12 lower expression in BM. Therefore, both Lr05V and Lr05NV treatments are able to activate beneficially the IL-17A/GM-CSF axis and accelerate the recovery of Cy-induced immunosuppression by increasing BM myeloid precursors. We demonstrated for the first time the beneficial effect of CRL1505 strain on myelopoiesis affected by a chemotherapeutic drug. Furthermore, Lr05NV could be a good and safe resource for reducing chemotherapy-induced leukopenia. The results are a starting point for future research and open up broad prospects for future applications of the immunobiotics.

## Introduction

Myelosuppression is the major dose-limiting toxicity of systemic cancer chemotherapy (1). Cyclophosphamide (Cy) is a widely used alkylating anti-cancer drug with a high therapeutic index and broad spectrum of antitumor activity (2, 3). Moreover, Cy induces dose-limiting suppression of proliferating hematopoietic progenitor cells and results in a marked neutropenia, but dose reductions could affect treatment efficacy (4, 5). At present, hematological rescue techniques, such as hematopoietic progenitor transplantation or administration of recombinant granulocyte colony-stimulating factor are applied to reduce the Cy-induced neutropenia (6–8). However, these treatments include new adverse effects, hospitalization fees, nosocomial pathogens exposition and pour life quality. Therefore, it is important to have safe agents capable of reducing myelosuppression and improving morbidity in patients undergoing chemotherapy. In recent years, probiotic lactic acid bacteria (LAB) has shown promising results in this regard.

Some immunomodulatory LAB can be a valuable tool to accelerate recovery from myelopoiesis and improve resistance to infections in immunosuppressed mice. We have previously shown that *Lactobacillus (L.) rhamnosus* CRL1505 increased resistance against infections in both immunocompetent and immunocompromised mice (9–11). The strain CRL1505 is able to accelerate the recovery of ontogeny B in bone marrow (BM) and spleen (10–13); potentiate basal myelopoiesis and improve emergency myelopoietic response in malnutrition-immunosuppressed hosts (13, 14). In addition, the preventive administration of *L. plantarum* CRL1506 to Cy-immunosuppressed mice increases resistance to opportunistic pathogens, as it improves BM myeloid cells and the innate immune response (15). The protective effects of immunobiotics depend on the administration way used, being the oral way the most efficient to potentiate myelopoiesis (16). However, the consumption of live microorganisms by immunosuppressed patients may represent a potential risk to their health. While the viability of the LAB is an important factor to achieve optimal beneficial effects, it is possible to stimulate immunity using non-viable LAB (17, 18).

The aim of this work is to study the ability of viable and non-viable immunobiotics to minimize myelosuppressive and immunosuppressive effects derived from chemotherapy.

## Materials and Methods

### Probiotic Lactic Acid Bacteria

*L. rhamnosus* CRL1505 and *L. plantarum* CRL1506 were selected because of their immunomodulatory capacity (9, 10, 16). These strains were obtained from the CERELA culture collection (Chacabuco 145, San Miguel de Tucumán, Argentina). The lyophilized strains were rehydrated in a medium containing peptone (1.5%), tryptone (1%), meat extract (0.5%) in distilled water at pH 7. The microorganims were cultured for 12 h at 37°C (final log phase) in Man-Rogosa-Sharpe broth (MRS, Oxoid). The bacteria were harvested by centrifugation at 3000 g for 10 min, washed three times with sterile 0.01 mol/l phosphate buffer saline (PBS), pH 7.2, and resuspended in sterile on PBS. Non-viable lactobacilli were obtained by exposition to ultraviolet radiation for 2 hours. The absence of bacterial growth was confirmed using MRS agar plates. Viable and non-viable LAB were harvested by centrifugation and washed with sterile 0.01 mol/l PBS, pH 7.2. Finally, bacteria were suspended in 10% non-fat milk to be administered to mice.

### Murine Model of Chemotherapy Induced Myelosuppression

6-week-old Swiss-albino male mice were obtained from CERELA. The myelosuppression was induced in all animals by intraperitoneal injection of Cy (150 mg/kg) on day 0 as previously described (15). During the 10-day study, immunosuppressed mice were housed in plastic cages, were kept in controlled environmental conditions with light dark cycles of 12 h, and were fed balanced conventional diet and sterilized water *ad libitium*. Researchers and special staff trained in animal handling and care were in charge of animal welfare, and monitored animal health and behavior twice a day. We strived to minimize the number of animals and their suffering. During the experimental period no signs of discomfort or pain were observed in the animals. Also, no deaths were reported before the mice reached end points. The CERELA Institutional Animal Care and Use Committee prospectively approved this study under the protocol CRL-BIOT-BCE-2013/2A, and all experiment comply with the current laws of Argentina and all international organizations for the use of experimental animals.

### Treatment of Cy-Induced Myelosuppressive Mice With LAB

**Figure 1:**
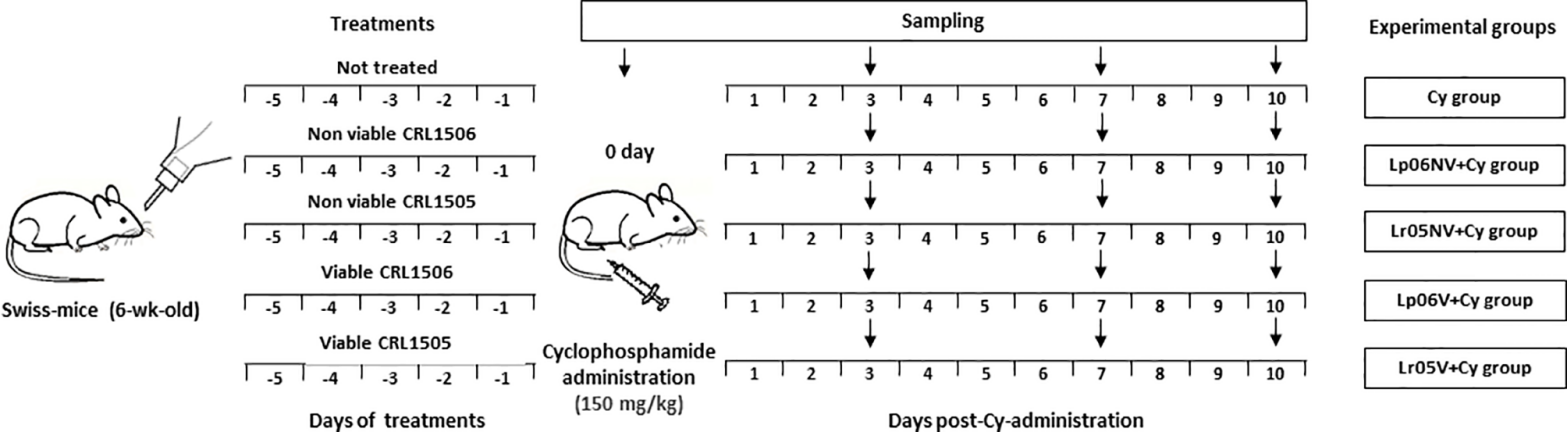
Schematic representation of the feeding protocols used in this study. Treatment of mice by feeding with viable or non-viable *L. rhamnosus* CRL1505 (Lr05V+Cy or Lr05NV+Cy groups) or *L. plantarum* CRL1506 (Lp06V+Cy or Lp06NV+Cy groups) for 5 consecutive days before injection of Cy. A control group (Cy group) were mice without probiotic treatment. Determinations were performed on day 0 (before Cy administration) and in different times after Cy administration for 10 days. The black down arrows indicate the sampling day. In the experiments 6 animals were used per group for each time point.

Different groups of mice were fed with 10^8^ cells/mouse/day of viable or non-viable *L. rhamnosus* CRL1505 (Lr05V+Cy or Lr05NV+Cy groups) or *L. plantarum* CRL1506 (Lp06V+Cy or Lp06NV+Cy groups) for 5 consecutive days before injection of Cy. These doses were previously selected as optimal for effects on the immune system (9). A control group (Cy group) consisted of animals that did not receive immunobiotic treatment (**Figure 1**). Six animals per group each time were used in the experiments. Determinations described below were performed on day 0 (before Cy administration) and in different times after Cy administration for 10 days.

### Histological Studies

The femoral bone from the different experimental groups was removed and immediately immersed in 4% paraformaldehyde, decalcified in 50% formic acid and 15% sodium citrate, and processed by standard histological techniques (paraffin-embedding) (13). 5 um sections from femurs were stained with Hematoxylin-Eosin.

### Total and Differential Leukocyte Count in Blood and BM

Blood samples were obtained from ketamine-xylazine-anesthetized animals by cardiac puncture in heparinized tubes. BM samples were obtained by flushing the femoral cavity with phosphate-buffered saline (PBS). A Neubauer counting chamber was used to count the blood and BM total number of leukocytes. The differential cell counts were carried out with smears stained with May Grünwald Giemsa under a light microscope (100x), and absolute numbers were calculated as described previously (15).

### Blood and BM Myeloperoxidase Activity

The myeloperoxidase (MPO) activity was used as a marker of myeloid cells in BM, and to evaluate functionality of myeloid cells in blood. Washburn test was used to measure the MPO activity. The results were expressed as percentages of peroxidase positive (Px+) cells (12).

### Isolation of BM Cells and Flow Cytometry

Single cell suspension from BM was prepared as previously described (16). 1 x 10^6^ cells were blocked with anti-mouse CD32/CD16 monoclonal antibody (BD Biosciences) and then stained with fluorescein isothiocyanate (FITC)-conjugated anti–mouse Ly6G antibody (clone 1A8, BD Biosciences); phycoerythrin (PE)-conjugated anti–mouse Gr-1 antibody (clone RB6-8C5, BD Biosciences), biotinylated-conjugated anti-mouse Ly6C antibody (clone AL-21, DB Biosciences); FICT-conjugated anti-mouse CD34 antibody (clone RAM34, BD Biosciences); biotinylated-conjugated anti-mouse Ly 6A/E antibody (Sca-1, clone D7, BD Biosciences); PE-conjugated anti-mouse CD117 antibody (C-kit, clone 2B8, DB Biosciences); FITC-conjugated anti-mouse TER-119/Erythroid cells antibody (clone Terr-119, BD Biosciences); and allophycocyanin (APC) mouse lineage antibody cocktail (clone 145-2C11). Following incubation with biotinylated primary antibodies, the labeling was revealed using streptavidin-PercP. The cells were acquired on a FASCalibur™ flow cytometer (BD Biosciences) and data were analyzed with FlowJo software (Tree Star).

### Apoptosis Assay

The BM cells of different experimental groups were harvested 12 h after Cy administration. Apoptosis of 1 x 10^6^ cells was determined with a FITC Annexin V Apoptosis Detection Kit I (BD Pharmingen). The suspension was analyzed by using FASCalibur™ flow cytometer (BD Biosciences) and data were analyzed with FlowJo software (Tree Star).

### Isolation of Lamina Propria Lymphocytes and Flow Cytometry

Lamina propria cells were obtained using the methodology of Deshmukh et al. (19) with modifications. Proximal ilea sections were removed from the different experimental groups on days 0, 3, 7 and 10 post-Cy, and minced into small pieces. Then, the cut tissues were incubated for 60 min with 300 U of Type I collagenase (Sigma-Aldrich) in 2 ml of RPMI 1640 medium (Sigma-Aldrich). The collagenase-treated minced tissues were gently grated into a plastic dish to obtain a single cell suspension, filtered and depleted of erythrocytes by hypotonic lysis (Tris-ammonium chloride, BD PharMingen). The cells were washed and resuspended in RPMI supplemented with 4% FCS. 1 x 10^6^ cells were blocked with anti-mouse CD32/CD16 monoclonal antibody (BD Biosciences) and then stained with fluorescein isothiocyanate (FITC)-conjugated anti-mouse CD335 antibody (NKp46) and biotinylated-conjugated anti-mouse CD4 antibody (BD Biosciences). Following incubation with biotinylated primary antibody, the labeling was revealed using streptavidin-PercP. For intracellular staining, the cells were fixed and permeabilized using Cytofix/Cytoperm and Permeabilization buffers (BD Biosciences) according to manufacturer instructions. Then, the cells were intracellularly stained with phycoerythrin (PE)-conjugated anti–mouse IL17A antibody and Alexa-conjugated anti–mouse RORγ antibody (BD Biosciences). The data were collected with a FASCalibur™ flow cytometer (BD Biosciences) and analyzed with FlowJo software (Tree Star).

### BrdU-Incorporation

The APC-BrdU-Flow-Kit (BD Pharmingen) was used for the analysis of BrdU-incorporation into bone marrow cells. After a single intraperitoneal injection of BrdU solution (1 mg/6g of body weight), BrdU was also administered at 1 mg/ml with drinking water for 3 days.

### Quantitative Expression Analysis by Real-Time PCR

We performed three-step real-time quantitative PCR to characterize the expression of CXCL12, GM-CSF, IL-1β, IL-3 and SCF mRNAs in BM. TRIzol reagent (Invitrogen) was used to isolate the total RNA from each sample. All RNA samples were quantified using Qubit^®^ RNA HS Assay Kit (Invitrogen) with the Qubit^®^ Fluorometer. All cDNAs were synthesized using a reverse transcription (RT) kit (SuperScript III First-Strand Synthesis SuperMix, Invitrogen) according to the manufacturer’s recommendations. Real Time qPCR was performed on an iQ5 Real-Time PCR Detection System (BioRad) with the IQTM SYBR^®^supermix (Bio-Rad) in 96-well plates. The following primers were used: CXCL12 (sense: 5’-GTC CTC TTG CTG TCC AGC TC-3’; antisense: 5’- TAA TTT CGG GTC AAT GCA CA-3’); GM-CSF (sense: 5’-CAT CAA AGA AGC CCT GAA CC-3’; antisense: 5’-TGC ATT CAA AGG GGA TAT CAG-3’); IL-1b (sense: 5’-GAC CTT CCA GGA TGA GGA CA-3’; antisense: 5’-AGG CCA CAG GTA TTT TGT CG-3’); IL-3 (sense: 5’-TAG GGA AGC TCC CAG AAC CT-3’; antisense: 5’-TTA GGA GAG ACG GAG CCA GA-3’); SCF (sense: 59-CGG GAA TCC TGT GAC TGA TAA-39; antisense: 59-GGC CTC TTC GGA GAT TCT TT-39). PCR was performed with 1 μL of cDNA or water in the non-template controls, 4 μL of primer mix (0.3 μM of each primer), 5 μL of RNase-free water and 10 μL of IQTM SYBR^®^ Green Supermix (BioRad). The PCR cycles consisted in: 95°C for 5 min, 40 cycles of 95°C for 30 sec, 55°C for 20 sec, 72°C for 30 sec and 95°C for 1 min. A melting curve analysis was performed immediately at the end of each experiment at a linear temperature transition rate of 0.5°C/sec from 55 to 95°C to determine the specificity of the amplification. The relative mRNA expression (as fold change) was determined relative to a positive control after normalizing to a housekeeping gene (β-actin) using the 2−ΔΔCT or Livak method (20).

### Serum IL-17 Measurement

The concentration of IL-17 was measured in serum samples from the different experimental groups on days 0, 3, 7 and 10 post-Cy by using commercially available cytometric bead array (CBA) technique, following the manufacturer’s recommendations (Mouse Flex Set, BD Bioscience, San Diego, CA, USA). Briefly, serum samples were diluted 1:2 with the assay diluent. The lyophilised standard was reconstituted in an assay diluent to have a standard curve. The capture beads were added to each tube (standard and samples). After incubation for 1 h at room temperature, PE detection reagent was added to each tube, followed by gentle mixing and incubation for 2 h at room temperature. After this, a wash buffer was added to each test tube before centrifuging. Finally, the supernatants were discarded and the wash buffer was added to resuspend the beads. Standards and samples were acquired on the BD FACSCalibur™ flow cytometer. The data were analyzed with the FCAP Array™ software (BD, Bioscience). The results were expressed in pg/mL using the standard curve performed with different concentrations of the cytokine.

### Statistical Analysis

GraphPad Prism software version 6.0 was used to perform statistical analysis. The results of three independent experiments were expressed as the mean ± SD. 2-way ANOVA was applied to normal distribution of data. The differences between the two groups were studied by comparing the means in pairs by Tukey test. Differences were considered significant at *P*<0.05.

## Results

### Effect of Lactobacilli on BM Tissue Damage Induced by Cy

**Figure 2:**
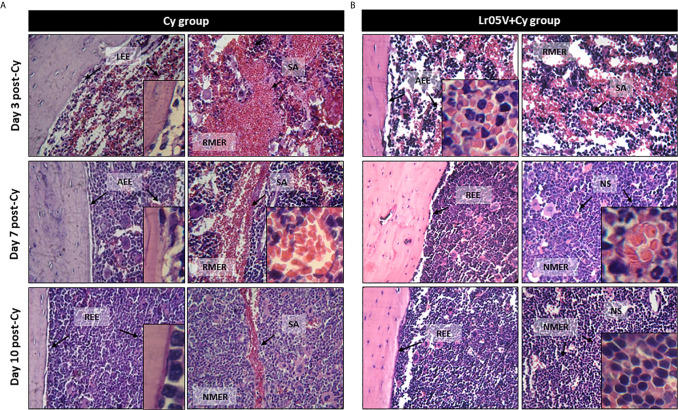
Histological examination of bone marrow architecture. The femurs were removed from control mice (Cy group) **(A)**, and mice treated with viable *Lactobacillus rhamnosus* (Lr05V+Cy group) **(B)** on days 3, 7 and 10 post-Cy administration. The samples were fixed in paraformaldehyde, decalcified in formic acid and sodium citrate, stained with hematoxylin and eosin and examined with a light microscope (400× and 1000× magnification). The results represent data from two independent experiments. Six animals were used for each time point by groups. LEE, Loss of endostal epithelium; AEE, altered endostal epithelium; REE, recovered endostal epithelium; RMER, reduced myeloid/erythroid ratio; NMER, normal myeloid/erythroid ratio; SA, sinusoidal alteration; NS, normal sinusoide.

It is known that Cy administration induced noteworthy alterations of BM histological characteristics (15). Thus, Cy-treated mice showed hipocellularity with reduction of the myeloid/erythroid ratio. In addition, sinusoidal alteration, increased numbers of mature red blood cells and fat cells, and loss of endostal epithelium were observed after Cy administration (**Figure 2A**). However, the mice treated with viable or non-viable lactobacilli showed fewer BM alterations than the Cy group during the early days after Cy treatment. Besides, recovery of tissue architecture was faster in the groups treated with viable *L. rhamnosus* CRL1505 (**Figure 2B**).

### Effect of Lactobacilli on BM and Blood Leukocytes Counts Induced by Cy

**Figure 3:**
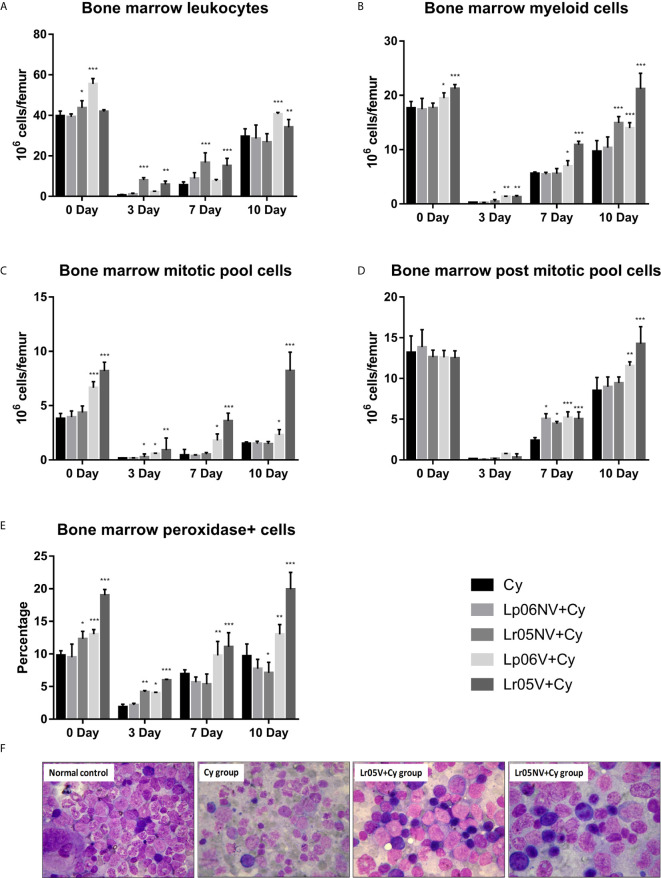
Bone marrow leukocytes. 6-week-old Swiss mice were fed with 10^8^ cells/mouse/day of viable or non-viable *Lactobacillus rhamnosus* CRL1505 (Lr05V+Cy or Lr05NV+Cy groups) or *L. plantarum* CRL1506 (Lp06V+Cy or Lp06NV+Cy groups) for 5 consecutive days before injection of Cy. The treated groups and the untreated control mice (Cy group) received one dose of 150 mg/kg Cy intraperitoneally. Bone marrow leukocytes **(A)**, myeloid cells **(B)**, mitotic pool cells **(C)**, post-mitotic pool cells **(D)** and peroxidase + cells **(E)**, counts were performed on day 0, before Cy administration, and days 3, 7 and 10 post-Cy administration. The results come from two independent experiments. Six animals per group each time were used in the experiments. Results are expressed as mean ± SD^. ***,**,*^ Significant differences from the Cy group should be as follows (p<0.0001, p<0.001 or p< 0.05 respectively). Photomicrographs of May Grünwald Giemsa-stained smears of bone marrow on day 3 post-Cy (Magnification X400) of normal control, and Cy, Lr05V+Cy and Lr05V+Cy groups **(F)**.

Cy administration induced a severe decrease in the total BM cell count at steady state, and the lowest number of cells was registered on day 3 post-injection of Cy. From day 7, an increase in the total cell count was observed, but did not reach normal values throughout the period studied (**Figure 3A**). Although on day 0 the studied groups presented similar baseline counts, the mice supplemented with Lp06V showed significantly higher values compared to the Cy group. In addition, those treated with non-viable and viable *L. rhamnosus* showed a less severe decrease in the total number of cells on day 3 post-Cy compared to the Cy group, and an earlier increase in the total number of cells observed on day 7 post-injection of Cy. Furthermore, the Lp06V+Cy and Lr05V+Cy groups reached normal values on day 10 post-injection (**Figure 3A**).

When morphologically recognizable elements were evaluated on day 0, before Cy injection, we observed that the preventive treatments with viable lactobacilli induced a significant increase in myeloid cells and mitotic pool compared to control (**Figures 3B, C**). However, no significant differences were observed in the post-mitotic pool cell count between the experimental groups (**Figure 3D**). Cy injection produced a significant reduction in the number of myeloid cells, mitotic and post-mitotic pools, reaching the greatest deterioration on day 3 post-injection, and the Cy group did not achieve normalization of these parameters in the period studied (**Figures 3B–D**). Opposite to this, the administration of viable lactobacilli induced a lower decrease in myeloid cell counts, mitotic, and post-mitotic pool cells on day 3 post-injection, and early recovery of these populations compared to Cy group (**Figures 3B–D**). In addition, the mice that received viable *L. rhamnosus* were able to normalize these counts in the period studied. Interestingly, the supplementation with non-viable *L. rhamnosus* showed a significant increase in the number of myeloid cells on days 3 and 10, and mitotic pool cells on day 3 compared to Cy group (**Figures 3B, C**).

The cytochemical study of the myeloid population in BM supported the morphological study since Lp06V, Lr05NV and Lr05V treatments induced an increase of the Px+ cell count in BM with respect to the Cy group before and after the Cy-administration (day 3) (**Figure 3E**). Furthermore, only viable lactobacilli treatment could normalize this parameter from day 7 post-Cy (**Figure 3E**).

**Figure 4:**
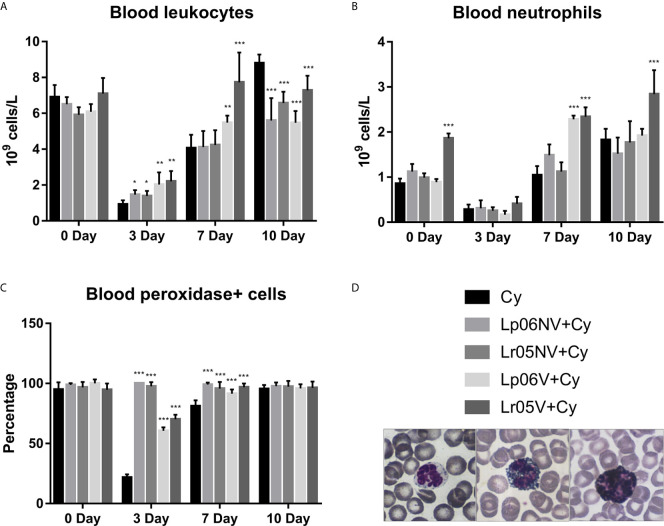
Blood leukocytes. 6-week-old Swiss mice were fed with 10^8^ cells/mouse/day of viable or non-viable *Lactobacillus rhamnosus* CRL1505 (Lr05V+Cy or Lr05NV+Cy groups) or *L. plantarum* CRL1506 (Lp06V+Cy or Lp06NV+Cy groups) for 5 consecutive days before injection of Cy. The treated groups and the untreated control mice (Cy group) received one dose of 150 mg/kg Cy intraperitoneally. Blood leukocyte **(A)**, neutrophils **(B)**, and peroxidase positive (+) cells **(C)** counts were performed on day 0, before Cy administration, and days 3, 7 and 10 post-Cy administration. The results come from two independent experiments. Six animals per group each time were used in the experiments. Results are expressed as mean ± SD. ^***,**,*^ Significant differences from the Cy group should be as follows (p<0.0001, p<0.005 or p<0.05 respectively). Photomicrographs of peroxidase+ neutrophils May Grünwald Giemsa-stained blood smears (Magnification X400) **(D)**.

Then, we studied the peripheral expression of Cy-induced changes in BM and the effect of preventive treatments (**Figures 4A–D**). Before Cy administration, leukocyte and Px+ cell counts showed no significant differences between the groups studied (**Figures 4A, C**). Regarding the number of neutrophils, the Lr05V+Cy group showed a significantly higher count compared to the Cy group (**Figure 4B**). Cy-administration induced marked leukopenia and neutropenia in all experimental groups, but with differences between them. Cy group mice showed the lowest values leukocytes and Px+ cells on day 3, with values 4 times lower than the basal value, reaching normalization on day 10 post-injection. In addition, neutrophil counts normalized from day 7 post-injection (**Figures 3A–C**). The groups treated with *L. rhamnosus* and *L. plantarum*, viable and non-viable, showed a less marked decrease in leukocyte and Px+ cell values on day 3 post-Cy compared to the control. No significant differences in the number of neutrophils were observed between the supplemented groups compared to the Cy group on day 3. The Lp06V+Cy and Lr05V+Cy groups were able to normalize the leukocyte and Px+ cell counts from day 7 post-injection, unlike the control, which normalized this parameter on day 10 (**Figures 4A, C**). Despite the fact that all the supplemented groups showed normal neutrophil values from day 7 post-injection, the Lp06NV+Cy, Lp06V+Cy and Lr05V+Cy groups achieved a significantly higher count of this parameter (**Figure 4B**).

### Effect of Lactobacilli on Myeloid Cell Maturation Impaired by Cy

**Figure 5:**
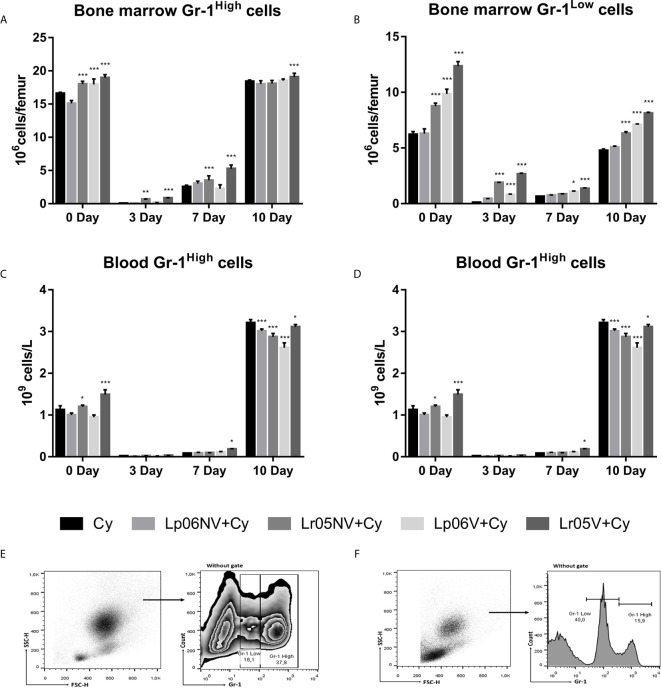
Gr-1 expression in bone marrow and blood by flow cytometry. 6-week-old Swiss mice were fed with 10^8^ cells/mouse/day of viable or non-viable *Lactobacillus rhamnosus* CRL1505 (Lr05V+Cy or Lr05NV+Cy groups) or *L. plantarum* CRL1506 (Lp06V+Cy or Lp06NV+Cy groups) for 5 consecutive days before injection of Cy. The treated groups and the untreated control mice (Cy group) received one dose of 150 mg/kg Cy intraperitoneally. Bone marrow Gr-1^High^ cells **(A)**, and Gr-1^Low^ cells **(B)**, blood Gr-1^High^ cells **(C)**, and Gr-1^Low^ cells **(D)** were performed on day 0, before Cy administration, and days 3, 7 and 10 post-Cy administration. The results come from two independent experiments. Six animals per group each time were used in the experiments. Results are expressed as mean ± SD. ^***,**,*^ Significant differences from the Cy group should be as follows (p<0.0001, p<0.002 or p< 0.02 respectively). Gating strategy to study Gr-1^High^ and Gr-1^Low^ cells in bone marrow **(E)**, and in blood **(F)**.

Next, we assessed the effect of different treatments with both viable and non-viable strains on the expression of Gr-1 cells (or a myeloid cell marker) in BM and blood. Before Cy-injection, Lr05V, Lr05NV and Lp06V treatments were able to increase the numbers of Gr-1^High^ and Gr-1^Low^ cells in BM. Only the mice that received the treatments with CRL1505 strain showed an increase of blood Gr-1^High^ cells. Additionally, blood Gr-1^Low^ cells in Lr06V+Cy and Lr05V+Cy mice were significantly higher than the Cy group (**Figure 5**). Cyclophosphamide induced an abrupt reduction of BM and blood Gr-1^High^ and Gr-1^Low^ cells on day 3 and 7 post-injection. However, Lr05NV+Cy and Lr05V+Cy mice showed a number of BM Gr-1^High^ cells on day 3 and 7 higher than the Cy group, as well as the number of BM Gr-1^Low^ cells on day 3. Only the viable CRL1506 strain had a similar effect on Gr-1^Low^ cells, comparable to the viable or non-viable CRL1505 strain (**Figure 5**). We decided to continue the assays of myeloid maturation in BM with the viable and non-viable *L. rhamnosus* CRL1505.

**Figure 6:**
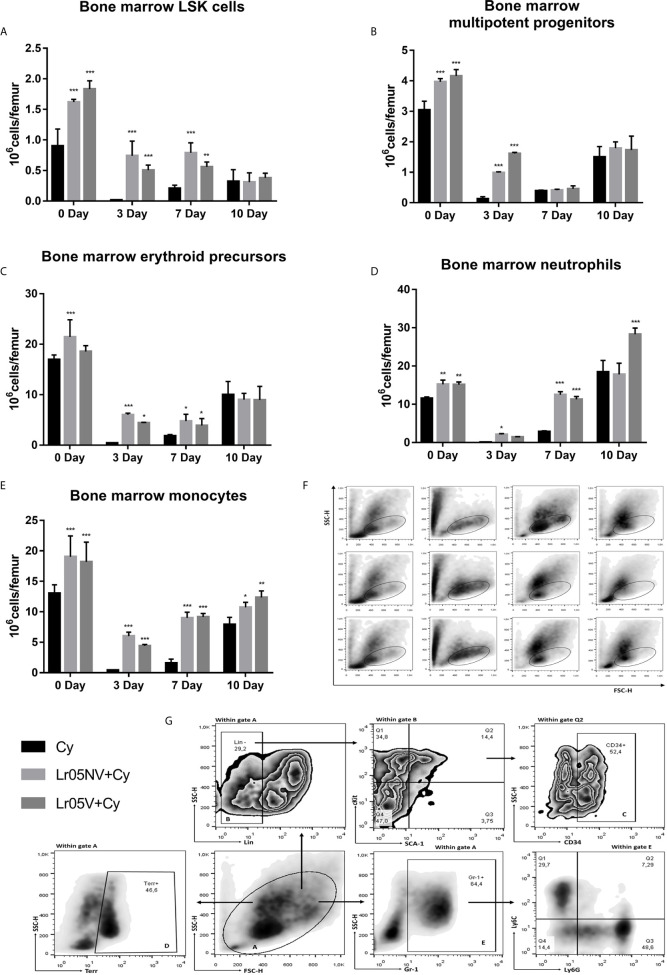
Myelopoietic precursors in bone marrow by flow cytometry. 6-week-old Swiss mice were fed with 10^8^ cells/mouse/day of viable or non-viable *Lactobacillus rhamnosus* CRL1505 (Lr05V+Cy or Lr05NV+Cy groups) for 5 consecutive days before injection of Cy. The treated groups and the untreated control mice (Cy group) received one dose of 150 mg/kg Cy intraperitoneally. Bone marrow LSK cells **(A)**, multipotent progenitors **(B)**, erythroid precursors **(C)**, neutrophils **(D)**, and monocytes **(E)** were performed on day 0, before Cy administration, and days 3, 7 and 10 post-Cy administration. The results come from two independent experiments. Six animals per group each time were used in the experiments. Results are expressed as mean ± SD. ^***,**,*^ Significant differences from the Cy group should be as follows (p<0.0001, p<0.002 or p< 0.02 respectively). **(F)** FSC vs SSC plots belong to days 0, 3, 7 and 10 post-Cy administration in the left-to-right columns, and to the Cy, Lr05V+Cy and Lr05NV+Cy groups in the top-to-bottom rows. **(G)** Gating strategy to study bone marrow hematopoietic stem cells (LSK) and myeloid linage cells: erythroid precursors, neutrophils and monocytes.

Lr05V y Lr05NV treatments were able to promoting the steady-state myelopoiesis in BM before Cy-injection. On day 0, viable and non-viable *L. rhamnosus* CRL1505 induced an increase of the number of hematopoietic stem cells (LSK cells: Lin^-^Sca-1^+^c-Kit^+^) (**Figures 6A, G**), multipotent progenitors (MPP: Lin^-^Sca-1^+^c-Kit^+^CD34^+^ cells) (**Figures 6B, G**), neutrophils and monocytes (**Figures 6D, E, G**) compared with the Cy control in BM. Cyclophosphamide impaired myelopoiesis by decreasing the number of different parameters studied in **Figure 6** mainly observed on day 3 and 7. Preventive administration of lactobacilli were equally effective in promoting early recovery of myeloid cells after Cy administration. Lr05V+Cy and Lr05NV+Cy mice showed higher counts of LSK cells and erythroid precursors (CD34^+^Gr-1^-^Terr119^+^ cells) on day 3 and 7, and MMPs on day 3 compared with the Cy group (**Figures 6A–C, G**). Regarding the mature cells studied, Lr05NV treatment induced an increase in neutrophils from day 3 post-Cy, while Lr05V from day 7 post-Cy (**Figure 6D**). Lr05NV and Lr05V mice showed monocyte numbers higher than the Cy group throughout the post-Cy period studied (**Figure 6E**).

### Effect of Lactobacilli on Hematopoietic Growth Factors and Cytokines Expression Impaired by Cy

**Figure 7:**
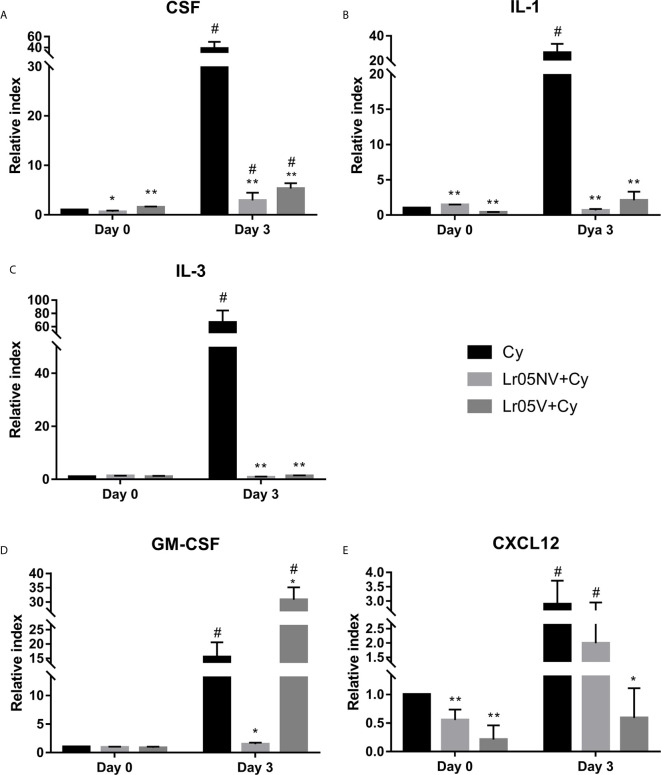
Expression of hematopoietic growth factors and cytokines in bone marrow. 6-week-old Swiss mice were fed with 10^8^ cells/mouse/day of viable or non-viable *Lactobacillus rhamnosus* CRL1505 (Lr05V+Cy or Lr05NV+Cy groups) for 5 consecutive days before injection of Cy. The treated groups and the untreated control mice (Cy group) received one dose of 150 mg/kg Cy intraperitoneally. Levels of mRNA of bone marrow CSF **(A)**, IL-1β **(B)**, IL-3 **(C)**, GM-CSF **(D)**, and CXCL12 **(E)** were performed on day 0, before Cy administration, and day 3 post-Cy administration. The results come from two independent experiments. Six animals per group each time were used in the experiments. Results are expressed as mean ± SD. ^**,*^ Significant differences from the Cy group should be as follows (p<0.0001 or p<0.0002 respectively). ^#^ Significant difference from the Cy group on day 0 should be as follows (p<0.0001).

Before Cy injection, both lactobacilli treatments significantly upregulated the expression of SCF, IL-1 and CXCL12 in the BM showing levels that were significantly higher than the Cy control, while IL-3 and GM-CSF expressions were not different from the Cy group (**Figure 7**). Cy-injection significantly increased the expression of SCF, IL-1, IL-3, GM-CSF and CXCL12 in BM of Cy group mice on day 3 (**Figure 7**). The expression of SCF, IL-1 and IL-3 in both Lr05NV+Cy and Lr05V+Cy mice was lower than in the Cy control on day 3 post-Cy (**Figures 7A–C**). In addition, Lr05NV+Cy mice showed a lower expression of GM-CSF, while Lr05V treatment increased the expression of this hematopoietic growth factor compared with the control (**Figure 7D**). Besides, mRNA levels of CXCL12 were significantly lower than Cy control mice in Lr05V+Cy group. However, the Lr05NV+Cy group CXCL12 expression was similar to the Cy group (**Figure 7E**). It is convenient to note that on day 3 the expression of the IL-1 and IL-3 in both Lr05NV+Cy and Lr05V+Cy mice was similar to the Cy control mice on day 0. Similarly, GM-SFC expression in Lr05NV+Cy and CXCL12 in Lr05V+Cy were not different from the Cy group mice before Cy-injection.

### Effect of Lactobacilli on Apoptosis and Proliferation of BM Cells During the Cy Treatment

**Figure 8:**
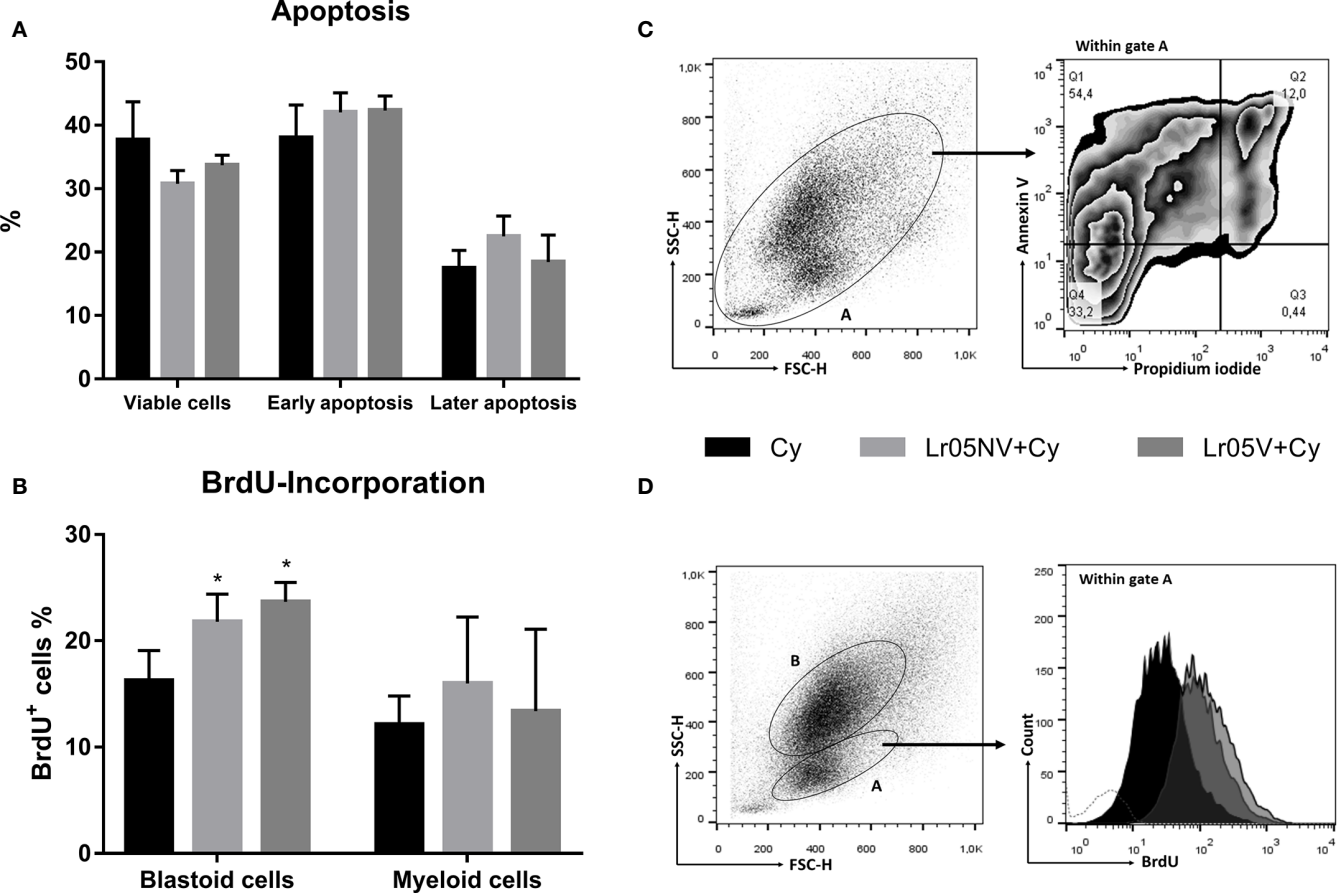
Apoptosis and cellular proliferation studies. 6-week-old Swiss mice were fed with 10^8^ cells/mouse/day of viable or non-viable *L. rhamnosus* CRL1505 (Lr05V+Cy or Lr05NV+Cy groups) for 5 consecutive days before injection of Cy. The treated groups and the untreated control mice (Cy group) received one dose of 150 mg/kg Cy intraperitoneally. **(A)** Apoptosis of bone marrow cells was determined by flow cytometry at 12 h post-Cy administration. **(B)** BrdU-incorporation into bone marrow cells was performed on day 3 post-Cy administration. The results come from two independent experiments. Six animals per group each time were used in the experiments. Results are expressed as mean ± SD. ^*^ Significant difference from the Cy group should be as follows (p<0.01). **(C)** The BrdU expression was analyzed by flow cytometry. **(C)** Gating strategy to study bone marrow viable cells (Annexina V-,Propidium iodide-), cells in early apoptosis (Annexina V+,Propidium iodide-), and cells in later apoptosis (Annexina V+,Propidium iodide+). **(D)** Gating strategy to study BrdU-incorporation in cells with blastoid morphology of bone marrow.

Next, we studied whether the immunoenhancing effect of the viable or non-viable *L. rhamnosus* was due to the inhibition of apoptosis induced by Cy, or due to an increase in the proliferation of myeloid precursors. Cyclophosphamide induced 60% apoptosis of BM cells at 12 h post-injection (**Figures 8A, B**). The preventive treatments with Lr05V and Lr05NV did not show significant differences in the percentage of apoptotic cells compared to the Cy control. Therefore, both treatments did not prevent apoptosis damage in BM cells (**Figure 8A**).

Then, we evaluated the lactobacilli influence on the proliferation of BM cells, through the incorporation of bromodeoxyuridine and subsequent detection by flow cytometry. The preventive administration with both viable and non-viable *L. rhamnosus* was able to increase significantly the incorporation of BrdU within BM cells with blastoid morphology compared with the Cy group (**Figures 8C, D**). No significant differences were observed when evaluating BrdU-incorporation in myeloid cells (**Figure 8C**).

### Role of the Intestinal Mucosa in the Immunopotentiating Effect of Myelopoiesis Induced by CRL1505 Strain

**Figure 9:**
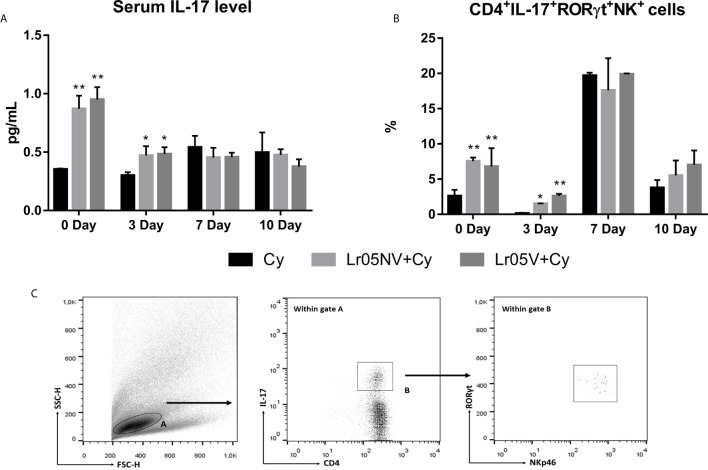
Serum Il-17A and group 3 innate lymphoid cells in the intestinal lamina propria. 6-week-old Swiss mice were fed with 10^8^ cells/mouse/day of viable or non-viable *Lactobacillus rhamnosus* CRL1505 (Lr05V+Cy or Lr05NV+Cy groups) for 5 consecutive days before injection of Cy. The treated groups and the untreated control mice (Cy group) received one dose of 150 mg/kg Cy intraperitoneally. Serum IL-17A level **(A)** and percentage of group 3 innate lymphoid cells in the intestinal lamina propria **(B)** were performed on day 0, before Cy administration, and days 3, 7 and 10 post-Cy administration. The results come from two independent experiments. Six animals per group each time were used in the experiments. Results are expressed as mean ± SD. ^**,*^ Significant differences from the Cy group should be as follows (p<0.002 or p<0.05 respectively). **(C)** Gating strategy to study CD4^+^IL-17^+^RORγt^+^NK^+^ cells in the intestinal lamina propria.

Finally, we study whether as a consequence of the interaction of viable or non-viable *L. rhamnosus* CRL 1505 with the intestinal mucosa, there is an increase in serum IL-17. We observed that the preventive treatments with Lr05NV and Lr05V induced serum IL-17A levels significantly higher than Cy control before Cy-injection and on day 3 post-Cy (**Figure 9A**). In addition, Lr05NV+Cy and Lr05V+Cy mice showed a greater ability to increase the percentage of group 3 innate lymphoid cells (ILC3s) (CD4^+^IL-17^+^RORγt^+^NK^+^ cells) in the intestinal lamina propria, since on day 0 and 3 post-Cy presented a percentage of ILC3s higher than the Cy group (**Figures 9B, C**).

## Discussion

In the last years there has been a large increase in the number of immunocompromised patients due to different causes. Among them, secondary immunodeficiency by chemotherapeutic treatments is one of the most common reasons of morbidity in cancer patients. Cancer is one of the principal causes of death around the world. In 2018, there were 18 million new cases and 9.6 million cancer-related deaths (21). As mentioned above, chemotherapeutics such as Cy have a high cytotoxicity on tumor cells. However, they also damage healthy tissues, this is why chemotherapy has important side effects (2–4).

Many attempts are being made to find safe immunoenhancing agents that reduce myelosuppression and enhance the immune response. For example, some plant extracts are known for their specific biological properties, which when administered to Cy-immunosuppressed animals are able to increase the immune response (22–24). Polysaccharides of different origins have also been described as protectors against myelosuppression and immunosuppression caused by Cy (25–28). There is currently a growing consumption of scientifically supported functional foods and health promoting dietary supplements (29). In this sense, probiotics have gained special interest (30) However, there is little literature of the probiotics effect on the recovery of the immune response impaired by treatment with immunosuppressive drugs. In this regard, it has been shown that a *L. plantarum* strain is capable of stimulating the proliferation of splenocytes of Cy-immunocompromised mice in response to lipopolysaccharide (31). However, Matsumoto et al. (32) demonstrated that the *Bifidobacterium longum* BB536 increases the resistance to sepsis caused by *Pseudomonas aeruginosa* in mice treated with Cy. Recently, other researchers demonstrated that both *L. plantarum* HY7712 and *L. casei* HY7213 can accelerate the recovery from Cy-immunosuppression, immunopotentiating NK cells and cytotoxic T lymphocytes, and restoration of the macrophages phagocytic capacity (5, 33). Leaving aside the research carried out by our group there is no other information about the potential effect of the administration of immunobiotics on BM, especially about the fact that they can stimulate the myelopoiesis or improve the immune-hematopoietic response in Cy-immunosuppressed hosts. Among the strains studied in our laboratory, we selected *L. rhamnosus* CRL1505 and *L. plantarum* CRL1506 for their efficiency to increase the resistance against infections in both immunocompetent and immunocompromised mice (9, 11). It is important to note that we chose oral administration of immunobiotics because it was more effective than nasal priming to enhance basal myelopoiesis and improve the emergency myelopoietic response in malnourished host (16).

The indisputable histological alterations of BM caused by Cy were reversed faster when the mice received a preventive treatment with viable or non-viable lactobacilli, being the Lr05V+Cy group which showed the most evident changes. The histological studies of BM during chemotherapy are poorly reported in the literature. However, similar results were only observed with preventive treatment with *L. casei* CRL431 (15), or with natural compounds derived from herbal plants, like Ginseng or *Rhus verniciflua* using different models of immunocompromised hosts (34, 35). Moreover, our histological results were correlated with the cell total counts and cell morphologically recognizable counts in both BM and blood. The treatments with Lr05V, Lr05NV and Lp06V were the most effective to induce the early recovery of leukocytes, myeloid cells, pool mitotic and post-mitotic cells and Px+ cells in BM. This recovery was reflected in peripheral blood. At the same time, these findings are consistent with those obtained by other experimental treatments used to restore myelopoiesis (5, 21, 31–33). Most of these studies showing a myelopoiesis-enhancing effect during chemotherapy, only showed morphologically recognizable cell counts, perhaps because they were used in the follow-up of patients during chemotherapy. However, we also demonstrated that Lr05V, Lr05NV and Lp06V treatments were able to improve the number of Gr-1^Low^ ​​cells, considered here as immature myeloid cells, and Gr-1^High^ cells as mature myeloid cells in BM and blood. Taking in to account the results described up to this point, we selected the strain CRL1505 for being the one capable of maintaining its myelopoiesis-enhancing properties in its non-viable form. Next, we deepened this study exploring the precursors of the myeloid lineage in BM.

It is known that the hematopoietic stem cells (HSC) are multipotent cells that reside in the BM and are characterized by a small set of lineage markers as Lin^-^Sca-1^+^c-Kit^+^ (LSK cells) (36). However, these phenotypically defined HSCs are heterogeneous in their potential for differentiation into progeny of multiple lineages and capacity for self-renewal (37, 38). A fraction of LSK cells gives rise to multipotent progenitors (Lin^-^Sca-1^+^c-Kit^+^CD34^+^ MPPs), which in turn can generate the megakaryocyte/erythrocyte lineage-restricted progenitor (Lin^-^Sca-1^-^c-Kit^+^FcγRII/III^-^CD34^+^ MEP) or the granulocyte/macrophage lineage-restricted progenitor (Lin^-^Sca-1^-^c-Kit^+^FcγRII/III^+^CD34^+^ GMP) (39). Our flow cytometry studies allowed to characterize LSK cells, MPPs, erythroid precursors (CD34^+^Ter119^+^ cells), polymorphonuclear precursors (CD34^+^Gr-1^+^Ly6G^+^Ly6C^-^ cells) and monocyte precursors (CD34^+^Gr-1^+^Ly6G^-^Ly6C^+^ cells) in Cy-immunocompromised mice. At this point, it is necessary to emphasize that the effect of Lr05V and Lr05NV treatments on myelopoiesis should be evaluated in the context of recovery kinetics. Thus, on day 0 and on the first post-Cy days, the preventive treatments mainly induced an increase of LSK cells and MPPs, without significantly affecting the number of more mature cells. At the end of the studied kinetics, the main effects of preventive treatments are evidenced on immature polymorphonuclear cells and monocytes. On the one hand, we can conclude that Lr05V and Lr05NV treatments are able to stimulate myelopoiesis at steady-state. At the same time these results were correlated with myelopoietic growth factors. Before Cy-challenge, both treated groups showed a high expression of CSF. On day 3, Lr05V+Cy group showed a high expression of GM-CSF. In addition, both treated groups showed low expression of CXCL12, an anchor sign of cells in BM. This would favor the exit of mature myeloid elements from BM and explain the improvement in the blood counts observed. Similar results were obtained with strain CRL1505 in the recovery of myelopoiesis in malnourished mice (16). On the other hand, these findings would be framed under the paradigm of the hierarchical differentiation of HSCs (39), opposing the idea of Sun et al. (40) and Busch et al. (41) that steady-state hematopoiesis is sustained mainly by progenitors rather than HSCs. While the consumption of live bacteria during chemotherapy is not recommended due to a possible risk to the patients’ health, our results support the treatment with the non-viable CRL1505 strain since it maintains the myelopoiesis-enhancement capacity of the viable strain. This strategy is hardly considered in the bibliography. It is known that the heat-treatment of bacteria disrupts the bacterial cell wall, inactivates protein and releases the cellular contents (42). Because the principal target of UV radiation is DNA, cell membranes of UV-killed cells retain not only the capacity to transport specific molecules acting as a permeability barrier, but also the capacity to maintain the electron transport *via* protein and lipid carriers and a residual cytoplasmic esterase activity (42). Therefore, in this work we used UV treatment to obtain non-viable immunobiotics. In previous studies we demonstrated that UV-killed *L. rhamnosus* CRL1505 was shown to be equally effective as the viable strain in increasing resistance to *S. pneumoniae* infection when administered nasally (18). This evidence is essential when it comes to immunocompromised hosts with high susceptibility to infections. However, the myeloenhancing effects of viable bacteria are much more intense than that of the non-viable *L. rhamnosus* CRL1505 when administered orally. We speculate that despite giving the mice exactly the same dose, the viable bacteria can resist gastrointestinal transit more successfully, interacting with the intestinal mucosa immunity more effectively than the non-viable bacteria. Surely, the UV-killed bacteria dose is lower at the intestinal lumen than the viable strain. An oral delivery system would be necessary to allow the non-viable bacteria to reach its action site unchanged.

Next, our purpose was to know the possible mechanisms of the myelopoiesis-enhancing effect of Lr05V and Lr05NV. We showed that even though preventive treatments do not modify the ability of Cy to induce apoptosis in BM cells, both increased the incorporation of BrdU *in vivo* in BM cells. This increase of proliferative capacity of BM cells allowed the early recovery of the proper balance between apoptosis and cell proliferation improving the homeostatic condition of HSCs in BM (43). Finally, another aspect considered was the increasingly relevant role of intestinal microbiota in the maintenance of hematopoiesis. It was demonstrated that the intestinal microbiota is necessary to maintain systemic neutrophils in the circulation, CD4^+^ T cells in the spleen, and HSCs in BM during steady-state myelopoiesis (44–46). The mechanisms by which commensal microorganisms can control the immune response at distant sites such as BM are not fully understood. It has been shown that the IL-23/IL-17/G-CSF axis participates in the regulation of myelopoiesis, and the neutrophil homeostasis in basal conditions depends on G-CSF, which is regulated through receptors of pattern recognition, linking the Toll-like receptor (TLR) with granulopoiesis (44). Microbiota derived components, such as LPS through TLR4/MyD88 signaling, induced intestinal IL-17 production and increased G-CSF plasma levels leading to granulocytosis (19). Here we demonstrate that viable and non-viable *L. rhamnosus* CRL1505 are able to increase the frequency of IL17A-producing innate lymphoid cells in the lamina propria of the small intestine (IL-17A^+^RORγt^+^CD4^-^NKp46^+^) after injection of Cy. These results were correlated with an increase in serum IL-17A levels. At the same time, at 3 days post-Cy, Lr05V treatment induced a high expression of GM-CSF and a lower expression of CXCL12 in BM, compared to the Cy group. Therefore, CRL1505 strain can beneficially activate the IL-17A/GM-CSF axis and accelerate recovery from Cy-induced immunosuppression by increasing the number of myeloid progenitors in BM.

We venture to propose that the use of myeloenhancing treatments with *L. rhamnosus* CRL1505 could reduce neutropenia without affecting the success of chemotherapeutic treatments, even regardless of the type of chemotherapeutic drug or tumor process. On the one hand, this is based on the fact that the immunobiotic CRL1505 treatments reinforce the physiological effects exerted by the intestinal microbiota by stimulating myelopoiesis as noted above. Furthermore, it is not ruled out that, when evaluating the effects of this strain in the context of a tumor process, it may have some anti-tumor effect per se, as has already been demonstrated for other immunobiotic LAB strains (47). On the other hand, the use of filgastrim is a common salvage therapy in different types of cancer. However, the role of filgrastim during acute myeloid leukemia (AML) induction therapy remains controversial (48). Patients diagnosed with AML who received filgastrim showed no significant difference regarding overall survival or progression-free survival compared with AML patients not treated with this drug. Furthermore, Abboud et al. (49) demonstrated that rG-CSF and selinexor can be combined safely in patients with relapsed or refractory acute myeloid leukemia. Moreouver, new studies using immunobiotic treatments in experimental cancer models could strengthen our results. The results shown here represent the starting point of a young line of research. We have an optimistic look at the possible application of these immunobiotics in patients undergoing chemotherapy, because of previous studies that demonstrated the ability to stimulate respiratory immunity in experimental animals and later humans (50).

In conclusion, we demonstrated for the first time the beneficial effect of *L. rhamnosus* CRL1505, an immunobiotic strain, on myelopoiesis affected by a drug used in chemotherapeutic treatments. Furthermore, we showed that although the viability of the LAB is an important factor to achieve an optimal effect, the administration of non-viable *L. rhamnosus* CRL1505 also induced early recovery from steady-state myelopoiesis in Cy-immunosuppressed hosts. The results of this work provide the scientific basis to propose non-viable *L. rhamnosus* CRL1505 as a new bioactive agent that enhances myelopoiesis. Its future application as a good and safe nutritional supplement in immunosuppressed pacients by chemotherapeutic drugs would make it possible to reduce the time required for the recovery of myelopoietic capacity and consequently the recovery of immunity. This would have an important impact on the management of these patients, since it would allow an optimal implementation of conventional therapy and improve their life quality. The results are a starting point for future research and open up broad prospects for future applications of the immunobiotics.

## Data Availability Statement

The original contributions presented in the study are included in the article/supplementary material. Further inquiries can be directed to the corresponding author.

## Ethics Statement

The animal study was reviewed and approved by this study was carried out in strict accordance with the recommendations in the Guide for the Care and Use of Laboratory Animals of the Guidelines for Animal Experimentation of CERELA. The CERELA Institutional Animal Care and Use Committee prospectively approved this research under the protocol CRL-BIOT-BCE-2013/2A. Researchers and special staff trained in animal handling and care were in charge of animal welfare, and monitored animal health and behavior twice a day. We strived to minimize the number of animals and their suffering. During the experimental period no signs of discomfort or pain were observed in the animals. Also, no deaths were reported before the mice reached end points.

## Author Contributions

SS and SA designed the study. AG, LS, EH, and SS did the experiments. SS, SA, and EH provided financial support. SS, SA, AG, and FG contributed to data analysis and results interpretation. SS and SA wrote the manuscript. All authors contributed to the article and approved the submitted version.

## Funding

This study was supported by grants of the Agencia Nacional de Ciencia y Técnica (PICT-2018-1093 and PICT-2018-3264) and CONICET (PIP272 and PIP531).

## Conflict of Interest

The authors declare that the research was conducted in the absence of any commercial or financial relationships that could be construed as a potential conflict of interest.
